# Profile of Liver Transplant Recipient in a Tertiary Hospital in Northern Spain

**DOI:** 10.3390/jcm12154934

**Published:** 2023-07-27

**Authors:** Janet Pagnozzi, Yuri Álvarez, Ignacio González-Pinto

**Affiliations:** 1 Área de Gestión Clínica de Geriatría, Hospital Monte Naranco, Avenida Doctores Fernández Vega 107, 33012 Oviedo, Spain; 2 Área de Teoría de la Señal y Comunicaciones, Universidad de Oviedo, Edificio Polivalente, Módulo 8, Campus Universitario de Gijón, 33203 Gijón, Spain; 3 Servicio de Cirugía General y del Aparato Digestivo, Hospital Universitario Central de Asturias, Avenida de Roma, 33011 Oviedo, Spain

**Keywords:** liver transplantation, retransplantation, age, indications of liver transplantation, parenchymal chronic liver disease

## Abstract

Spain has the highest rates of liver transplantation (LT) per million inhabitants in the world, with the profiles of both donors and recipients in Asturias, a region in northern Spain, being different from the rest of the country. The main goal of this study was to carry out a preliminary analysis of the characteristics of LT recipients in Asturias, as well as of the basic characteristics of surgery and the postoperative period, and to discuss whether the results obtained in this study were comparable to what is described in the literature. This was a retrospective, descriptive, cross-sectional study, analyzing the LT carried out in a reference center of Asturias between 2002 and 2017. Relative and absolute frequency distributions for qualitative variables are provided, as are position and dispersion measures for quantitative variables. Using the multivariate Cox regression model, the prognostic factors associated with overall survival were determined. A total of 533 LTs were analyzed; 431 were men and 102 were women. The mean age was 55.1 years, concentrated between 40 and 69 years for both genders. LT was performed for chronic parenchymal liver disease (mostly of alcoholic etiology) and the recipients underwent surgery in an advanced stage of liver disease. Of these recipients, 8.1% (43 patients) were retransplantions, 65.1% in the first year due to primary graft dysfunction and complete hepatic artery thrombosis. Most patients had presented a grade II of Clavien−Dindo as the most frequent complication. Biliary complications were found in 12.3% of patients, with the main cause of death in the first 30 days being instability in the 24 h after LT. The median survival of the group was 13 years, with a 5-year survival probability of 79.3% and a 10-year survival probability of 61.9%. In view of the analyzed series, it can be concluded that the most frequent recipient profile was a male patient (mean age 55 years), with a significant alcohol habit, who was overweight, with chronic parenchymal liver disease of alcoholic or viral etiology, and who had reached the Child C stage before LT. This study could lay the foundations for future studies, to complete this analysis with the characteristics of LT surgery, its postoperative period, and the follow-up after discharge, to obtain a broader view of LT recipients in this region.

## 1. Introduction

Liver transplantation (LT) is indicated in patients with acute or chronic liver diseases when other therapeutic alternatives have been exhausted and when the estimated life expectancy is less than one year. Thanks to technological and scientific advances, LT is becoming a widely used and accessible procedure.

The beginnings of LT coincided with the development of vascular surgery techniques by A. Carrel. Half a century later, the first experimental LT in dogs were described by V. Staudacher in 1952 [1]. F. Moore and T. Starzl described a series of replacements in animals [1,2], and the first human patient to survive LT surgery was transplanted by Starzl in 1963. In Europe, the start of LT was due to the work of R. Calne and Roger Williams who reported five cases of LT in 1968. The pioneering research of Starzl and Calne in the field of LT was awarded with the 2012 DeBakey Award for Clinical Medical Research [1].

The first solid-organ transplant in humans was performed in 1954, when a successful kidney transplant was performed between identical twins [3]. However, although the benefits of LT in different pathologies was observed, it was not possible to carry out the first liver transplant in humans until the appearance of effective immunosuppressive drugs and preservation solutions [4].

Between 1968 and 1979, Starzl et al. published 170 cases of LT. In those days, the survival rate per year was 32%, as mortality from infections and/or uncontrolled rejections was very high and there were multiple side effects due to the required medication. Besides, liver grafts do not tolerate hot ischemia well, and there were no liver-support techniques, such as kidney dialysis.

Studies and series published in the 1960s made possible improvements in donor selection, due to the understanding that the liver parenchyma was extremely sensitive to circulatory changes in the donor, and to the methods of preservation and subsequent conservation of the organ [5]. To this end, the works of Schalm et al. [6], Brettschneider et al. [7], and Slapak [8], who combined hypothermic continuous perfusion and the use of hyperbaric oxygen, can be cited, as can the works of Brown [9], who studied preservation by cooling and dehydration and Moss [10] who used cooling from −20 °C to −60 °C. These works contributed to improving the success in terms of survival and functionality of the organ. Furthermore, the discovery of the so-called Wisconsin’s solution made possible the exchange of organs between distant regions, thanks to its long preservation time (12 to 18 h) [10], compared to 2.5–3 h of the first preservation solutions [6]. 

The acceptance of the concept of brain death in the United States in 1968 was an additional milestone for LT, since it allowed the preservation of donor organs in ideal physiological conditions, which resulted in better graft quality and survival, as was the introduction of immunosuppressants such as cyclosporine and tacrolimus [1]. These immunosuppressants allowed less toxicity and better prevention of rejection and serious opportunistic infections compared to azathioprine, as well as additional improvements in survival [11,12].

With fewer than 10 LT centers in the world, the number of LT performed in Europe until 1980 barely reached 20. A milestone for LT happened in June 1983, when the first consensus conference of the National Institute of Health (NIH) concluded that liver transplantation was a valid therapeutic modality for end-stage liver disease. From this date, there was a rapid expansion of LT centers worldwide, and by the end of 1988 approximately 1000 LT were performed each year both in the USA and in Europe.

Spain has the highest LT rates per million inhabitants in the world [13]. Thus, an adequate selection of candidates, as well as a clear definition of indications and contraindications, is of paramount importance. The latter are dynamic and can vary from one center to another, reflecting the personal experience and research interests of each LT program. 

In this article, the authors present an analysis of the characteristics of LT recipients in Asturias, a region in northern Spain characterized by its aging society [14], as well as of the basic characteristics of surgery and the postoperative period. In particular, higher average age and life expectancy, as well as a higher prevalence of chronic diseases together with the changing epidemiology of liver disease, would result in an increase of the age of both LT candidates and donors [15,16], considering that the latter does not generate major complications. Furthermore, these factors would help reduce mortality on the waiting list and the shortage of available organs [16]. We also compare the results obtained in this study with those identified in the literature, and discuss whether they are similar or not.

## 2. Materials and Methods

### 2.1. Study Design and Study Population

This was a retrospective, descriptive, cross-sectional study of all cases of brain-dead liver donors carried out in a tertiary level hospital in the north of Spain (Central University Hospital of Asturias, HUCA, located in Oviedo, Spain) between April 2002 and December 2017. It involved the review of 533 medical records. The HUCA is the reference center for liver surgery and liver transplantation for the Principality of Asturias, a region with a population of 1,004,686 [14]. The inclusion criteria were: patients who underwent the complete LT intervention at the HUCA, with age greater than 16 years, and who had signed the informed consent. Patients who died during surgery, without completing surgery, were excluded as well as those for whom, after a reasonable search, it was not possible to obtain clinical information.

A data collection protocol was designed. It included the selected clinical−pathological parameters that could have a useful value when performing the analysis of the data. The qualitative variables considered for the analysis were:

- Sex of the recipient;
- Cause of LT;
- Clinical history of interest of the recipient;
- Presence of arterial variants in the donor, presence of hemodynamic instability during the surgical act and in the first 24 h after it;
- Need for arterial graft;
- Presence of reperfusion syndrome;
- Result of Menghini needle biopsy;
- Doppler ultrasound;
- Need for liver retransplantation during admission (if so, indicating the cause);
- Mortality/survival 30 days after surgery;
- Patient’s condition at the end of the study;

The quantitative variables registered for the analysis were:

- Age of the recipient and the donor at the time of transplantation;
- Child classification at the time of inclusion of the patient on the surgical waiting list for liver transplantation;
- Body mass index of the recipient;
- Duration of the surgery (considered to be from the skin incision to its closure);
- Use of blood products (number of packed red blood cells);
- Time of cold ischemia and hot ischemia;
- Flow in the hepatic artery and in the recipient’s portal vein;
- Occurrence of postoperative complications during admission (based on the Clavien−Dindo scale);
- Length of hospital stay (in days).

### 2.2. Statistical Analysis

Relative and absolute frequency distributions for qualitative variables, and position and dispersion measures for quantitative variables are provided. Univariate survival analyses were performed with Kaplan−Meier survival curves. Using the multivariate Cox regression model [17], the prognostic factors associated with overall survival were determined. The statistical analysis was carried out using the statistical software R, version 3.6.3 [18].

## 3. Results

### 3.1. Recipient Profile

**Figure 1:**
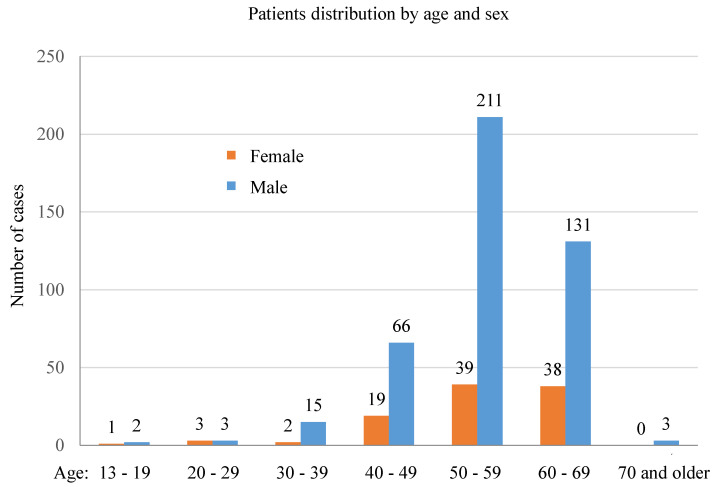
Distribution of the patients by age and sex.

Of the 533 LTs performed in the studied period, 431 patients (80.9%) were male, while 102 (19.1%) were female. The mean age of this series was 55.1 (±8.7 years), being 55.2 (±8.4 years) for males and 54.7 (±9.9 years) for females. Figure 1 shows the distribution by age and sex, in which it can be observed that the majority of patients were between 40 and 69 years of age (males, 94.6% and females, 94.1%). Of the male patients, 94.7% were within the 40–69 year age group, and this percentage was almost the same for the female patients (94.1%). Thus, this age group represented, without distinction of gender, the largest number of transplants performed (504 transplants out of 533).

**Figure 2:**
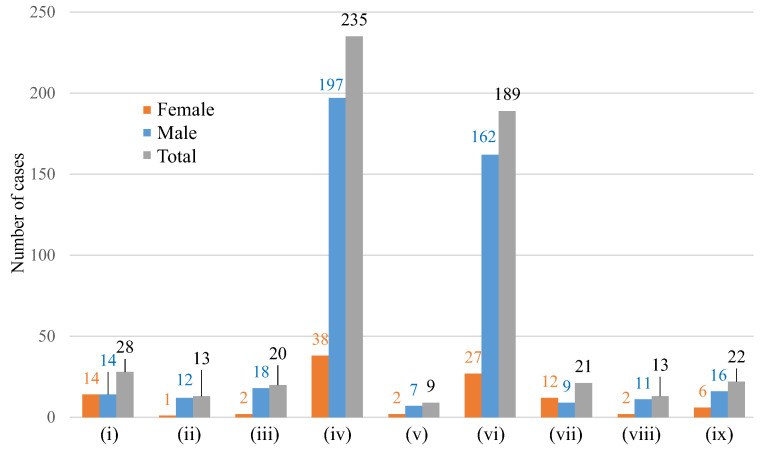
Indications of LT by type of pathology: (**i**) cholestatic chronic liver disease, (**ii**) chronic vascular liver disease, (**iii**) biliary-tree vascular disease, (**iv**) parenchymal chronic liver disease, (**v**) neoplastic disease, (**vi**) parenchymal liver disease plus neoplastic disease, (**vii**) acute and/or subacute liver failure, (**viii**) metabolic and/or genetic disease, and (**ix**) other causes (e.g., graft failure or thrombosis).

Indications of LT by type of pathology are summarized in Figure 2. Parenchymal chronic liver disease (424 cases ((iv) + (vi) in Figure 2) corresponding to 79.5% of total cases) stand out, followed by neoplastic disease (192 cases, ((v) + (vi) in Figure 2), 37.2%). Other pathologies were present in fewer than 6% of the cases: cholestatic chronic liver disease (5.3%), chronic vascular liver disease (2.4%), biliary-tree vascular disease (3.8%), acute and/or subacute liver failure (3.9%), and metabolic and/or genetic disease (2.4%). Other causes such as graft failure, thrombosis, or incompatibility made up 4.1% of the cases. 

Different indications of LT, such as hepatocellular carcinoma on cirrhosis, may coexist in the same patient. In these patients, hepatocellular carcinoma has been considered as an indication for transplantation. In this study, 59.3% of transplant patients had a history of previous alcoholism; however, LT due to alcohol cirrhosis alone was only been indicated in 26.1% of cases. 

The most frequent pathology and habits associated at the time of transplantation for this study were:

- History of previous alcoholism: 316 patients (59.3%);
- Smoking prior to or at the time of transplantation: 245 patients (46.0%);
- Kidney disease with creatinine levels > 1.5 mg/dL: 46 patients (8.6%);
- Diabetes mellitus: 141 patients (26.5%);
- Hypertension: 73 patients (13.7%);
- Heart disease: 41 patients (7.7%);
- Previous major abdominal surgery—both that performed at the hepatic level and at another level of the gastrointestinal tract: 16 patients (3.0%);
- Portal thrombosis, both partial and complete: 5 patients (0.9%).

It should be noted that several patients had more than one associated pathology. In particular, 180 (33.8%) patients had two pathologies, 80 (15.0%) patients had three pathologies, and 29 (5.4%) patients had three or more pathologies.

**Figure 3:**
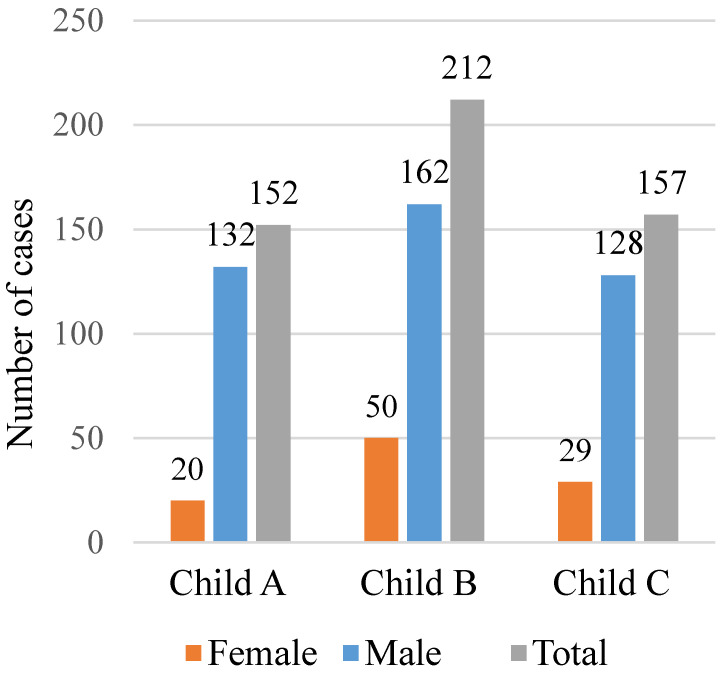
Distribution of LT patients according to Child classification.

Most patients had LT in advanced stage of liver disease, the majority at Child C or Child B stage (39.8% and 29.5% of cases, respectively), with 28.5% at Child A stage, as shown in Figure 3.

Out of 533 recipients, 43 patients were retransplanted, representing 8.1% of the total transplant patients in the period studied. Of these, 28 patients (65.1%) were retransplanted during the first year, 8 (18.6%) during the second year, 3 (7.0%) during the third year, and the remaining 4 (9.3%) after the third year. 

The main causes of retransplantation during the first year were:

- Primary graft dysfunction: 7 cases (25.0% of the total of 28 patients);
- Complete hepatic artery thrombosis: 7 cases (25.0%);
- Ischemic cholangiopathy: 6 cases (24.4%);
- Complete portal vein thrombosis: 4 cases (14.3%);
- Inferior vena cava stenosis, acute refractory graft rejection due to AB0 incompatibility, HCV recurrence, and recurrent cholangitis: 1 case (3.6%) each.

According to the body mass index (BMI) of the patients, 326 patients were overweight and 203 were normal weight; 9 patients were underweight with a BMI below 18.

### 3.2. Surgery Profile

**Figure 4:**
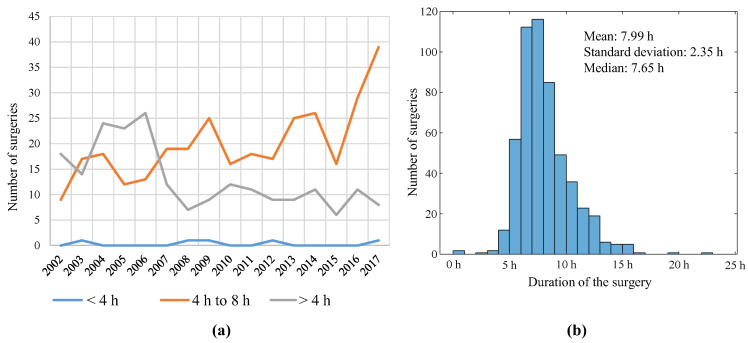
(**a**) Duration of the surgeries per year and (**b**) distribution of the duration of the surgeries.

An analysis of the duration of LT surgeries and their evolution over the years was conducted. Figure 4a shows the duration of surgeries, where it can be noticed that the number of surgeries whose duration was greater than 8 h has reduced since 2008. The number of surgeries requiring between 4 h and 8 h has increased in recent years, which is due to the increase in the number of transplants. The distribution of the duration of LT surgeries is shown in Figure 4b, with the average duration of surgeries being 8 h.

Mortality in surgeries was also analyzed by number of cases. Table 1 shows that the mortality decreased as more surgeries were performed, and was below 5% in the last 133 surgeries (7 out of 133). It also shows the correlation between the duration of surgeries and the mortality: surgeries lasting more than 8 h usually correspond to complications, which explains why the for these surgeries mortality was higher than for surgeries lasting between 4 h and 8 h. In the case of these shorter surgeries, the mortality rate was reduced when comparing the first 300 surgeries with the final 233.

With regard to the use of blood products, in this series most patients received transfusion of packed red blood cells (517 patients, 96.9%); only 3% received no transfusions (16 patients). Of those who received packed red blood cells, 62.6% (324 patients) received fewer than 10 units, while 37.3% (193 patients) received more than 10 units.

A total of 69 patients presented instability, with 35 also showing instability at 24 h. Reperfusion syndrome occurred in 173 patients (32.4%), while for 360 (67.5%), it did not.

Only one anastomosis was performed in most patients (433 cases, 84.7% of the 511 LTs for which this information was available). In 66 out of 511 (12.9%) two anastomoses were performed, and only in 12 cases (2.3%) were more than two performed. The reason why some patients required more than one arterial anastomosis was the presence of arterial anatomical variants (in all the cases, the variant was right hepatic artery from superior mesenteric artery) or the technical need to use a graft due to the length of the arterial ends, in order ensure that there was no tension in the anastomosis. The use of grafts in this series was 2.4% (13 cases).

**Figure 5:**
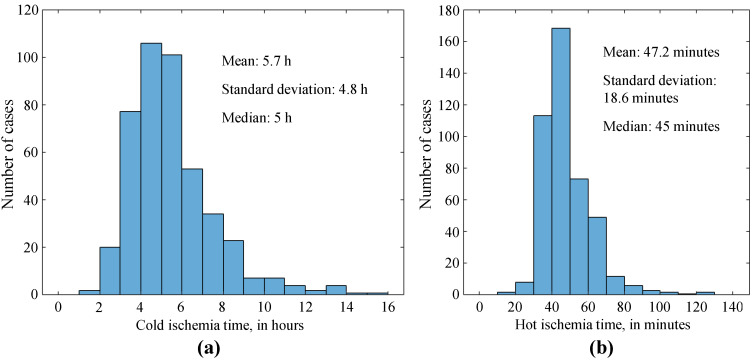
Distribution of cold ischemia (**a**) and hot ischemia (**b**) times.

The average preservation time of the organ was 5 h and 39 min for cold ischemia, and 47 min for hot ischemia. The distribution of cases according to the times of cold ischemia and hot ischemia is shown in Figure 5. No changes in the trend for cold ischemia were observed over the years, whereas the number of transplants with hot ischemia greater than 1 h has decreased over the years. It is necessary to point out that there was a lack of records of ischemia time (both cold and hot) in the first years of LT at the HUCA, with ischemia time data missing 90 and 93 times for cold and hot ischemia, respectively, out of a total of 533 transplants.

Regarding vascular flows, the mean flow for the portal vein was 2.1 L/min while the mean flow for the hepatic artery was 278.2 mL/min. Data could not be obtained for 210 LTs in the case of portal blood flow and 188 LTs in the case of arterial blood flow due to data-registration issues and the fact that there was no flow meter when the transplantation unit at the HUCA was created.

### 3.3. Post-Surgery Data Analysis

**Figure 6:**
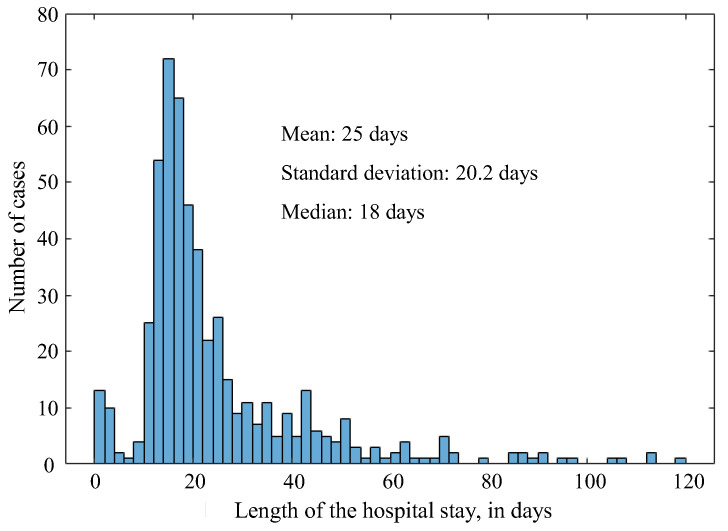
Distribution of hospital stay time.

The average hospital stay in this series was 25 days. As shown in Figure 6, most stays lasted from one to three weeks. No significant change of the hospital stay was observed over the years. For the 43 that were retransplanted, 27 (62.8%) stayed between one and three weeks, and the remaining 16 (37.2%) stayed more than three weeks.

Apart from analytical analysis, which was performed on 100% of patients, the main diagnostic study performed on transplant patients was hepatic Doppler echo. Of the 533 performed, data were available for only 437 LTs. This was due to failure in the collection of data in the clinical history and the fact that the Doppler echo technique was not systematically available for the first LT cases. 

The following diagnostic−therapeutic investigations were also conducted: (i) thoraco−abdominal computed tomography (CT) was performed for 207 out of the 533 cases (38.8%); (ii) angio-CT, 103 cases (19.3%); (iii) endoscopic retrograde cholangiopancreatography (ERCP), 40 cases (7.5%); (iv) magnetic resonance cholangiography, 45 cases (8.4%); and (v) Kehr trans cholangiography, 55 cases (10.3%). Other less frequent tests used as diagnostic studies of the hepatic process (5 cases or less) were fistulography, percutaneous angioplasty, transcholecystic cholangiography, magnetic resonance angiography, diagnostic arteriography and transjejunal cholangiography. In other cases, due to the postoperative course of the recipient, it was necessary to perform a therapeutic gastroscopy, a skin biopsy, and/or and a lung scan.

As part of the postoperative studies, liver biopsy was performed during the postoperative period in 123 patients (23%). The most common finding of biopsies was grade I acute rejection (38 cases), followed by grade II acute rejection (32 cases), and thirdly, signs of pericholangitis.

The complications arising in the postoperative period were collected using the Clavien−Dindo classification. For this series, the number of cases for each category was:

- Grade II: 304 patients (57.0%);
- Grade IIIA: 66 patients (12.3%);
- Grade IIIB: 74 patients (13.8%);
- Grade IVA: 25 patients (4.6%);
- Grade IVB 4 patients (0.75%);
- Grade V: 60 patients (11.2%).

It can be observed that, in this series, most patients had a grade II Clavien−Dindo classification. These were patients who required pharmacological treatment other than grade I (i.e., deviations from the normal postoperative period that do require open and/or endoscopic reoperations, use of blood transfusions or blood products, or parenteral nutrition). This is consistent with the value of the average hospital stay (Figure 6).

The analysis of the relationship between the length of hospital stay and the Clavien−Dindo classification is summarized in Table 2. It can be seen that 52% of transplant patients with a Clavien−Dindo II classification were hospitalized for 12 to 18 days. If Clavien−Dindo II patients hospitalized for 12 to 24 days are added, this percentage rose to 74.7%. Another conclusion drawn from Table 2 is that, of the 60 patients with Clavien−Dindo V classification, 40% were hospitalized fewer than 6 days (rapid death) and 28.3% for more than 30 days (death due to complications after transplantation).

Another analysis of interest is the survival rate and number of Clavien−Dindo V patients per age group, which is shown in Table 3. It can be observed that the percentage of Clavien−Dindo V patients increased with age (8.2% in the 40–49 age group; 12.4% in the 60–69 age group).

Those patients who were retransplanted (43 out of 533) had a survival rate of 50%, slightly below the survival rate of the series, which was 66%. The number of Clavien−Dindo V patients that were retransplanted was 6 (14%).

### 3.4. Analysis of Vascular and Biliary Complications

The relationship between several variables collected in the database and their possible correlation with the presence of vascular complications in patients was analyzed. Due to small sample size or number of cases in some categories, portal flow and donor age have been omitted from the analysis. 

In the results of the multivariate analysis, it can be observed that, when the causes of liver transplantation are “other causes” (Figure 2) such as graft failure, thrombosis, incompatibility, or chronic rejection, the probability of the risk of vascular complications increases. Two other variables that reached statistical significance were the time of hot ischemia (the longer the time of hot ischemia, the greater the risk of vascular complications), and the presence of anatomical alterations such as dissection of the recipient’s intima (the presence of these alterations also increased the risk of vascular complications).

Of the 533 patients, 66 (12.3%) exhibited biliary complications. The distribution of these 66 cases was the following:

- Biliary stenosis: 31 cases (46.9% of all complications);
- Bile leaks: 25 cases (37.8%);
- Combination of biliary leak and stenosis: 9 cases (13.6%);
- Accidental exit of the Kehr tube: 1 case (1.5%).

It was observed that, when the causes of LT were acute or subacute liver failure, the risk of biliary complications was significantly increased. The other two variables that reached statistical significance were the CHILD classification (patients with CHILD B or C classification showed lower risk of having biliary type complications), and those patients with biliary-tract alteration (greater risk of suffering biliary complications).

### 3.5. Mortality and Survival Analysis

The main factor that was significantly associated with the risk of dying in the first 30 days after liver transplantation was to the presence of instability in the first 24 h after surgery. The other two variables that reached statistical significance in the multivariate model were: (i) The length of stay: if the stay was longer than 7 days, the probability of death was lower. This was because patients with complications usually died in the first days after transplantation. (ii) Patients having chronic parenchymal liver disease (of 235 patients, 15 died in the first month after transplantation) or chronic parenchymal liver disease and neoplastic disease (of 189 patients, 12 died in the first month after transplantation).

**Figure 7:**
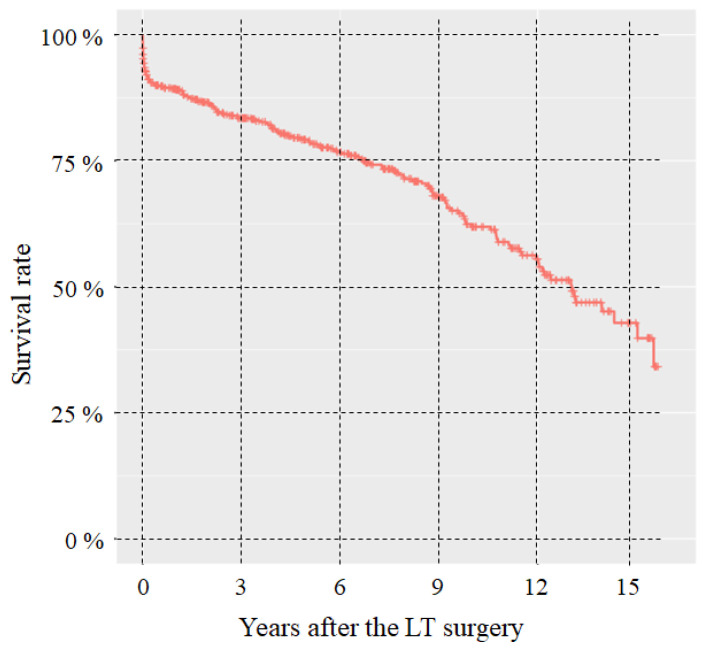
Survival rate after the LT surgery.

The survival rate was analyzed by defining the follow-up time as the difference between the date of transplantation and the date of *exitus*, if it occurred, or the closing date of the present study (31 December 2017). There are 158 *exitus*, with 13 years of median survival. The 5-year survival rate was 79.3%, decreasing to 61.9% at 10 years. The survival rate after the LT surgery is depicted in Figure 7.

Next, Cox regression models were constructed to determine the factors associated with overall survival. The variables cold ischemia time, hot ischemia time, and the presence of bile-duct alterations after the Doppler of the first month were omitted, as they could not be considered statistically significant. The results obtained from the statistical analysis showed that, when the cause of transplantation was biliary-tree vascular disease, the risk of exitus increased (hazard ratio, HR, of 2.65, *p* < 0.05). Patients who present instability during the first 24 h after transplantation (HR = 3.07, *p* < 0.05), and/or whose MELD was greater than 30 (HR = 3.51, *p* < 0.05), also had a higher risk of *exitus*.

## 4. Discussion

Indications for LT have varied over time, so they could be extended to include pathologies in which LT was not previously considered a valid treatment option. Other indications, such as viral cirrhosis due to HVB and HVC, have decreased. This increase in some indications has created an imbalance between the supply and demand of liver grafts, increasing the waiting list, as well as the average time spent by recipients in it [19].

In Spain, it has been observed that there has been a progressive increase in the rate of organ donors since 2014 compared to other countries and Spain also has one of the highest rates of liver transplant activity in the world. A study published in 2008 by Bruna et al. [20] pointed out this fact. In particular, Ref. [20] compared the results of two different periods of LT in the same Spanish hospital, observing that the number of donations, as well as the average age of donors, had been increasing over time. Other aspects to be assessed in Asturias through future studies would be the cause of death of the donors as well as their pathological antecedents, so this can be correlated with the rest of the country.

According to 2021 data from the Spanish National Transplant Organization (ONT) [21], the country has one of the highest transplant recipient rates per million inhabitants in the world, only behind the United States. This fact is also supported by the study presented in [22], where it is stated that “Spain has the highest rate of transplants per population due in large part to its highly efficient organ procurement system”.

In Asturias, specifically, according to the latest data, there is one of the highest rates of donors per million people nationwide, as well as effective donors in brain death. This results in mortality on the waiting list being one of the lowest due to the short time that recipients remain on it [21].

LT is a procedure that generates a high consumption of both human and material resources. Thus, the procedure and protocol to be followed must be optimized to obtain the best results with the minimum possible expense [23]. According to studies carried out in Spain, it is evident that there was a high use of blood products, although thanks to new anesthetic monitoring techniques, this consumption shows a decreasing trend [24].

The economic cost of LT, as well as the impact that the MELD score can have on the pre-transplant phase, is the basis of some studies [25], where the use of autologous liver grafts created by bioengineering from stem cells has been studied as a way to reduce the economic costs of a pathology. It is expected that liver diseases will increase in coming years compared to the trend of decreasing donors that has been observed in recent years [25].

These premises will be the basis for future studies at a regional level in Asturias, to assess the impact of LT on the public regional health system. An adequate selection of recipients prior to the inclusion in a waiting list for LT also influences the achievement of good results, optimizes resources, and generates a lower impact on the health system.

The series analyzed in the present study was a large one for the period studied (2003–2017) when compared with studies carried out in other Spanish hospitals [26,27]. The data obtained from the analysis of the results have allowed a clear profile of the LT recipient in Asturias to be drawn. This can be summarized as follows: a middle-aged male (predominantly alcoholic) who is overweight or has some degree of obesity, with advanced liver disease at Child C or Child B stage. This profile is consistent with the fact that for these patients LT is the last existing therapeutic option. The percentage of retransplantation was low, being 8.1% and occurring, in most cases, in the first year after the surgery. Although this figure is above the national average, it is still below that of some Spanish groups, as indicated in [28]. 

Another finding on this series was that the most frequent cause of retransplantation was vascular nature, in agreement with other studies [28]. Other causes of retransplantation also involved complete thrombosis of hepatic artery, ischemic cholangiopathy, or complete thrombosis of the portal vein. 

Most patients (504 transplants out of a total of 533) were concentrated in the age group between 40 years and 69 years, with both genders quite evenly represented. These results differ slightly from the data published at a national level by the Spanish ONT [21], in which it is evident that the age of recipients in the age group of 60 to 69 years has been increasing, while the group of 45 to 59 years has remained stable. According to the 2019 report of the Spanish ONT [19], which analyzes the different regions of the Spanish territory that have LT units, the evolution of the age of LT recipients on the whole country waiting list is quite homogeneous, with a decreasing trend over the years.

Similar to what was commented by Cuervas-Mons et al. in the *Analysis of the Spanish LT Registry* [26] and that reported by Schleicher et al. [27], the main indication of LT by disease group has been chronic parenchymal liver disease, specifically alcohol cirrhosis. In the studies presented in both [20,26] (the latter analyzed an older period and in a single center), it was observed that the median age of the LT recipient in Spain increased until reaching 59 years in the period 2010–2012, with the most frequent indications being, as in this series, liver cirrhosis and hepatocellular carcinoma.

With regard to LT surgery, it can be concluded that, since the beginning of activities of the LT unit of the HUCA, the duration of the interventions has been reduced. Surgeries lasting more than 8 h have been decreasing, inversely proportional to those lasting between 4–8 h, with a progressive decrease in the mortality of patients who underwent surgery. This can be justified by the learning curve of the surgeon and the LT team. This conclusion is partially supported by the study published in [29], stating that since its implementation until today, LT units have already consolidated their training in LT. Nevertheless, LT it is a subspecialty of general surgery with high technical difficulty and little distribution at the Spanish national level. According to [29], there are few specialists in training who want to dedicate themselves to LT in the future. This could be another future approach for LT research in Asturias and that would contribute to the improvement of results. 

One of the main limitations of this research was the lack of data about the donors in the clinical history, which makes it difficult to correlate the information about patients and donors, so the donors have not been analyzed in the present study. It would also be of interest to analyze specific data from the patient’s postoperative period such as intensive-care-unit stay and data inherent to post-discharge follow-up, which will be taken into account in future research.

## 5. Conclusions

In this contribution, the authors present a profile of the transplant patient in the region of Asturias (northern Spain) where HUCA is the reference hospital for LT. Statistical analysis was conducted in order to outline the basic characteristics of the transplant patient in this region, performing a comparison with other series, and aiming to establish improvement strategies. The characteristics of the recipients and their post-transplant survival statistics were analyzed, confirming that, although the age of recipients and their comorbidity is increasing, patients receiving LT are still of productive age, which generates high costs for the health system in particular and for the society in general.

Further studies will be devoted to completing the analysis of the series presented in this contribution by taking into account the type of immunosuppressive regimen received by the patients, and the analysis of the postoperative stay in the intensive care unit (not just the overall hospital stay). It will be of interest to complete the database with the missed data about the donors so this information can be correlated with the existing data on the recipients. 

## Figures and Tables

**Table 1:** Analysis of mortality in surgeries per 100 cases.

No. of Surgeries	% of Mortality	Mortality as a Function of the Duration of the Surgery		
		Number of Cases		
		<4 h	4 h to 8 h	>8 h
1 to 100	15.0	0	5	10
101 to 200	10.0	0	4	6
201 to 300	17.0	2	9	6
301 to 400	11.0	1	4	6
401 to 500	5.0	0	1	4
501 to 533	6.1	1	0	1

**Table 2:** Relationship between the length of the stay and Clavien−Dindo classification.

Clavien−Dindo	Length of the Stay (in Days)					
	<6	6–12	12–18	18–24	24–30	>30
II	1	25	158	69	23	28
IIIA	0	1	13	15	16	21
IIIB	2	1	13	15	10	33
IVA	0	0	2	2	5	16
IVB	0	0	0	1	0	3
V	24	3	8	4	4	17

**Table 3:** Mortality and survival of LT patients by age group. The percentage is calculated over the number of patients per age group.

Age Group (Years)	Number of Patients per Age Group	Clavien−Dindo V	Survival Rate
13–19	3	0 (0%)	3 (100%)
20–29	6	1 (16.7%)	3 (50%)
30–39	17	4 (23.5%)	11 (64.7%)
40–49	85	7 (8.2%)	52 (61.2%)
50–59	250	27 (10.8%)	170 (68.0%)
60–69	169	21 (12.4%)	110 (65.1%)
70 and older	3	0 (0%)	3 (100%)

## Data Availability

The database with the patients’ information cannot be disclosed due to privacy restrictions.
